# MRI of the fetal heart: comparison of the obstetric sonography and fetal magnetic resonance imaging findings

**DOI:** 10.1186/1532-429X-18-S1-O117

**Published:** 2016-01-27

**Authors:** Flavia P Junqueira, Taisa D Gasparetto, Heron Werner

**Affiliations:** 1 Radiology, DASA-SP, Sao Paulo, Brazil; 2 Radiology, DASA-RJ, Rio de Janeiro, Brazil

## Background

Intra uterine diagnosis of cardiac malformations is very important because they are associated with high morbidity and mortality. Despite advances in sonographic equipment, fetal position, maternal obesity, maternal abdominal wall scar from previous surgery and olygohydramnios, can impair significantely the visualization of fetal cardiac structures. The intent of this study was to evaluate the feasibility of fetal magnetic resonance imaging (MRI) with steady-state free precession (SSFP) sequence to visualize the normal and pathological appearances of the fetal cardiovascular system, comparing with the ultrasound (US) images.

## Methods

All patients were evaluated with MRI (1,5 T) and US at the same day, and had at least 26 weeks of gestation (mean gestational age: 30 weeks). N: 60 hearts. Patients were divided in two groups. Group 1: 55 control hearts with normal USG findings. Group 2: 5 hearts with US suspicion of congenital heart disease (CHD) including 1 case of conjoined twins, 1 case of transposition of the great vessels, 1 case of right chambers enlargement and ectasy of the venous coronary sinus and 1 case of Fallot Tetralogy. In both imaging techniques we evaluated morphological and functional aspects of the fetal heart. At analysis of MR images, all cardiac anatomic components were classified into one of two categories: well visualized, poorly or nonvisualized. In each group, the Student's t test was used to assess the relation between visibility of fetal cardiac features and gestational age. A value of p < 0.05 was considered to indicate a statistically significant difference.

## Results

MRI of the heart along the three fetal body planes (axial, coronal, and sagittal) was successful in all cases in the study. The left and right ventricular outflow tracts were identified in their corresponding transverse views in 49 (81,7%) and 38 (63,3%) fetuses, respectively. The inflow veins (superior and inferior venae cavae) were identified in 58 fetuses (96,7%) all cases. MRI of the fetal heart showed good anatomical correlation with the US images in the normal fetuses. In 3 of the pathologic cases, interventricular defects were successfully identified (Fallot, conjoined twins and transposition of the great vessels). MRI also showed good correlation in the pathologic cases, being superior to US in the conjoined twins case, as the US failed to demonstrate the correct anatomy of the two hearts.

## Conclusions

Fetal MRI with SSFP sequences in static and cine-resonance acquisition may help in the evaluation of the normal fetal heart and identification of the main morphological alterations related to CHD, when the US examination is limited by the fetal position, maternal obesity, and oligohydramnios. Additionally, both obstetric US and fetal MRI presented good correlation of findings and can be used as adjunct imaging techniques.

**Figure 1:**
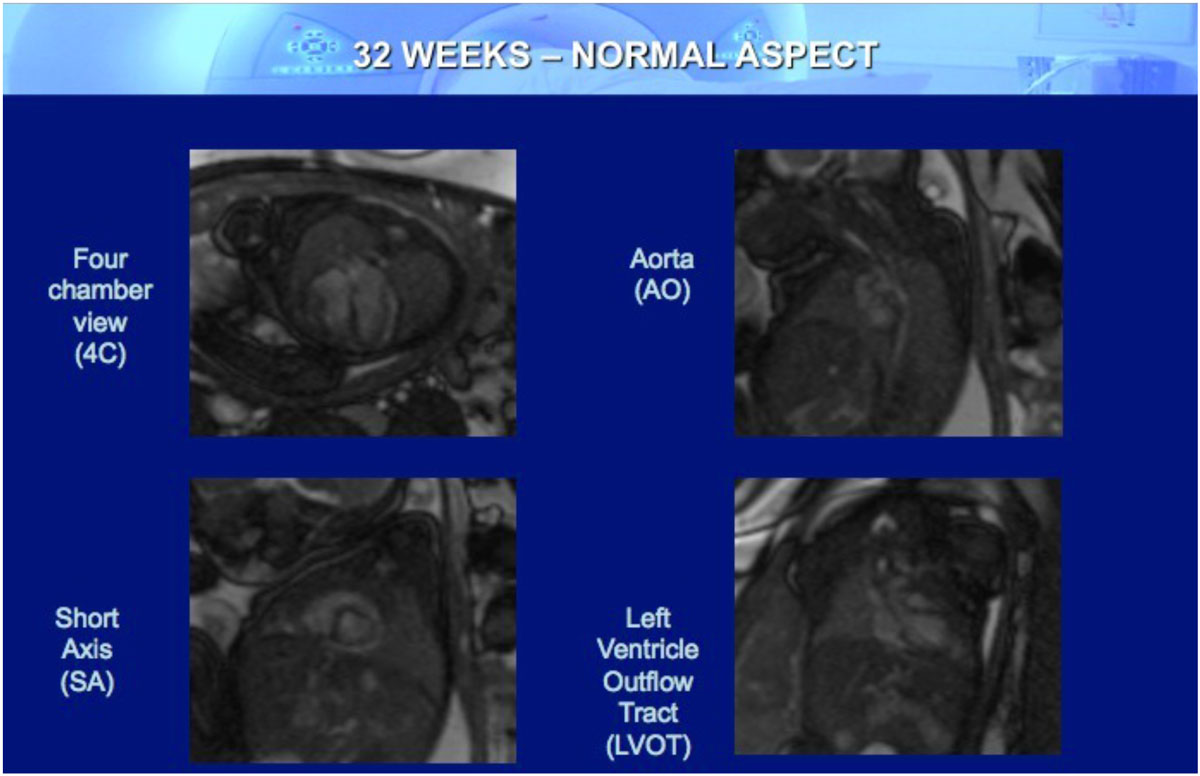
**Fetal CMR showing the normal aspect of a 32 weeks fetal heart showing four chamber and short axis views, left ventricle outflow tract and thoracic aorta**.

**Figure 2:**
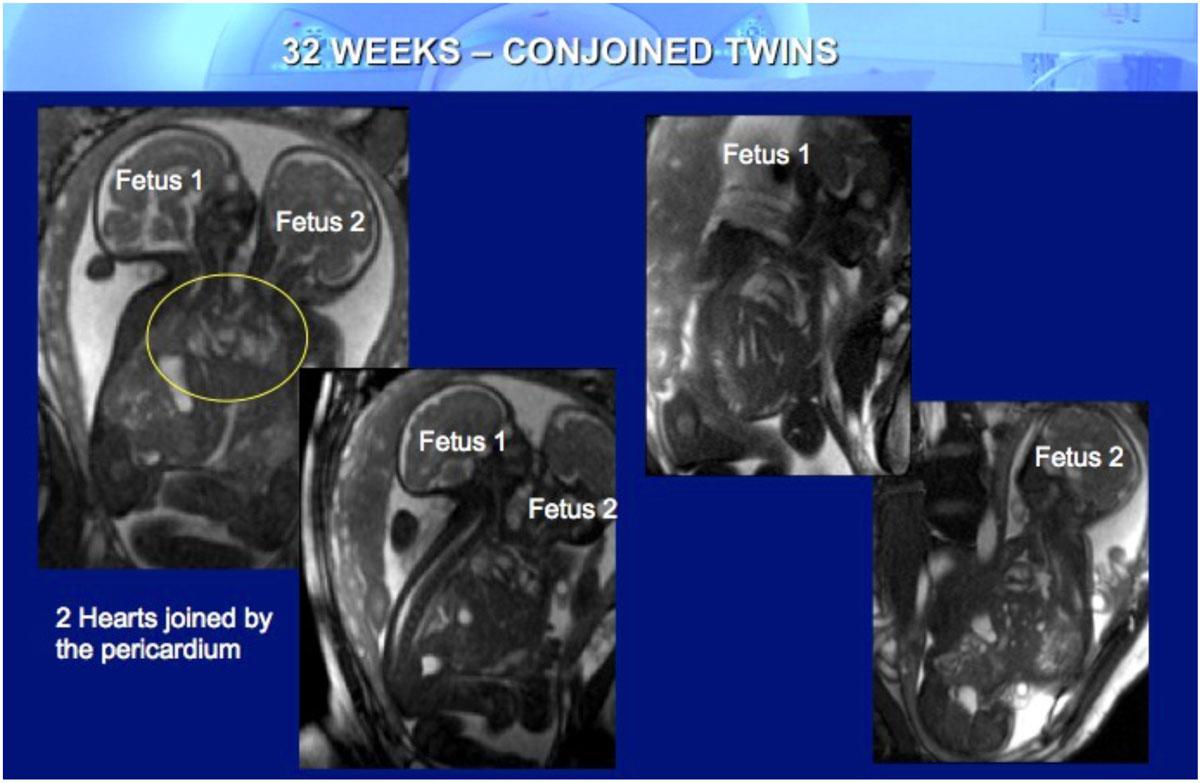
**Fetal CMR showing images of 32 weeks conjoined twins with the two hearts joinej by the pericardium**.

